# Circ_0092012 knockdown restrains non-small cell lung cancer progression by inhibiting cell malignant phenotype and immune escape through microRNA-635/programmed death ligand 1 axis

**DOI:** 10.1080/21655979.2022.2080386

**Published:** 2022-06-19

**Authors:** Jin Yan, Jian Zhu, Xiaoli Zhu, Hailing Liu, Guoping Chen

**Affiliations:** a Department of Respiratory and Critical Care Medicine, Binhai County People’s Hospital, Yancheng, Jiangsu, China; b Department of Respiratory and Critical Care Medicine, Zhongda Hospital Southeast University, Nanjing, Jiangsu, China; c Department of Radiology, Binhai County People’s Hospital, Yancheng, Jiangsu, China

**Keywords:** Circ_0092012, miR-635, PDL1, NSCLC, apoptosis, immune escape

## Abstract

Circular RNAs have been reported to play roles in non-small cell lung cancer (NSCLC) progression. Herein, this work aimed to investigate the potential value of circ_0092012 in NSCLC progression. Levels of genes and proteins were detected using quantitative reverse transcription-polymerase chain reaction and Western blot, respectively. The growth, malignant phenotypes and immune escape in NSCLC were investigated. The binding between microRNA (miR)-635 and circ_0092012 or programmed death ligand 1 (PDL1) was verified. Circ_0092012 was highly expressed in NSCLC. Circ_0092012 deficiency suppressed NSCLC cell proliferation, invasion and migration, moreover, as well as was able to inhibit the apoptosis of CD8 + T cells and induce higher interferon-γ and tumor necrosis factor-α levels when co-cultured with peripheral blood mononuclear cells. Mechanistically, circ_0092012 sponged miR-635, which targeted PDL1. Further rescue experiments suggested that the anticancer effects of circ_0092012 knockdown were reversed by miR-635 inhibition. Additionally, miR-635 re-expression suppressed NSCLC cell malignant phenotypes mentioned above and immune escape, which were attenuated by PDL1 overexpression. Moreover, circ_0092012 deletion retarded NSCLC growth *in vivo*. In all, circ_0092012 knockdown suppressed NSCLC cell oncogenic phenotypes and immune escape by miR-635/PDL1 axis.

## GRAPHICAL ABSTRACT

**Figure:**
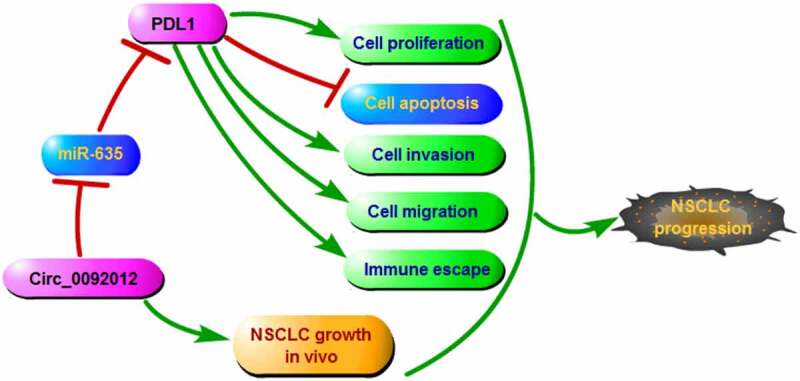

## Highlights

- Circ_0092012 knockdown restrained NSCLC malignant phenotype and immune escape.
- Circ_0092012 acted a sponge for miR-635 to up-regulate its target PDL1.
- Circ_0092012 performed its effects via miR-635/PDL1 axis.

## Introduction

Lung cancer accounts for the most common cause of mortality through the world, and approximately 85% of new cases are non-small cell lung cancer (NSCLC) [1]. There are about 1.6 million deaths annually [2]. Despite the advancement in clinical management and survival prolongation, many NSCLC patients cannot escape eventual metastasis, recurrence, and chemoresistance [3,4]. Therefore, further elucidation of the mechanisms underlying NSCLC malignancy is necessary.

Circular RNAs (circRNAs) are characterized as a kind of RNAs with a circular structure [5]. CircRNAs show sequence conservation and high stability in eukaryotes with cell/tissue/developmental-stage specific feature [6,7]. Studies have revealed that circRNAs are implicated in a wide variety of cell biological processes, such as apoptosis, proliferation, immune response and cellular stress response [8–10]. Moreover, extensive findings reported the abnormally expressed circRNA in distinct types of malignancies, including lung cancers, and deregulated circRNAs plays an essential function in cancer pathological process [11–14]. As example, CircRNA ZNF609 accelerated the growth and metastasis of thyroid cancer by down-regulating miR-514a-5p [15]. Besides, Zhang *et al*. showed that circ_0001287 inhibits the proliferation, metastasis, and radiosensitivity of NSCLC by miR-21/phosphatase and tensin homolog axis [16]. Circ_0092012 is originated from the exon 9 to 15 of the filamin A (FLNA) gene in chrX: 153592389–153594592, which was higher in laryngeal squamous cell carcinoma, and promoted cancer cell migration [17]. However, the action of circ_0092012 in NSCLC progression remains vague.

It has proposed that some circRNA can regulate gene expression by sponging microRNA (miRNA/miR) to prevent miRNA-mediated degradation of the messenger RNA (mRNA), thereby exerting its biological functions [18,19]. MiRNAs are a family of small non-coding RNAs of approximately 22 nucleotides. Emerging evidence has reported that miRNAs are able to participate in tumorigenesis and progression of various cancers, including NSCLC [20–22]. miR-635 is a well-recognized tumor suppressive miRNA in many of cancers, such as gastric cancer [23], osteosarcoma [24], as well as NSCLC [25]. However, the upstream regulators of miR-635 in NSCLC remain poorly understood. Programmed death ligand 1 (PDL1) is one of the ligands of PD-1 that is widely expressed in healthy cells, besides, the binding of PD-1/PDL1 induces immune escape and accelerates T-cell tolerance by reducing CD8 + T-cell and effector function [26,27]. PDL1 was expressed in NSCLC, and PD-L1-positive expression was frequently associated with worse postoperative prognosis in NSCLC patients [28]. Inhibition of PD-1/PDL1 could prevent the tumorigenesis and progression of NSCLC [29,30]. In this study, preliminary bioinformatics analysis indicated that PDL1 and circ_0092012 share the same microRNA response elements of miR-635. However, the relationship among miR-635, PDL1 and circ_0092012, as well as whether miR-635 and PDL1 mediate the action of circ_0092012 in NSCLC remains unclear.

Hence, we hypothesized that circ_0092012 might be involved in NSCLC progression. Then, the functions of circ_0092012 in NSCLC tumorigenesis *in vitro* and *in vivo* were investigated. Therefore, we also explored whether miR-635/PDL1 acted as the underlying axis of circ_0092012 to involve in the effects of circ_0092012 on NSCLC growth and progression.

## Material and methods

### Human samples

NSCLC tissues and adjacent normal tissues were collected from 27 NSCLC patients newly diagnosed by pathological and clinical examinations, and then preserved at −80°C until use. This study was allowed by the Ethics Committee of Binhai County People’s Hospital, and all subjects had signed written consent forms.

### Cell lines and cell culture

NSCLC cell lines H460, H1299 and A549, normal HBE cells and the 293 T cells were obtained from Chuan Qiu Biotechnology (Shanghai, China), and then cultured in the Dulbecco’s Modified Eagle Medium (DMEM) plus 10% fetal bovine serum (FBS) and 1% antibiotics with 5% CO_2_ at 37°C.

### Reverse transcription and quantitative reverse transcription-polymerase chain reaction

Total RNA was prepared using TRIzol reagent. Then, complementary DNAs (cDNAs) were synthesized using Geneseed® II First Strand cDNA Synthesis Kit with Random or Oligo(dT)18 primers, and the levels of circ_0092012 and PDL1 were detected using the SYBR® Green Supermix Kit (Takara, Dalian, China). Besides, the AMV Reverse Transcriptase (Solarbio, Beijing, China) and SYBR Premix Ex Taq II (TaKaRa) were employed for reverse transcription and qRT-PCR for miR-635. The relative gene expression was detected by the 2^−ΔΔCt^ method [31]. The primers are listed in Table 1. Actinomycin D (Solarbio) was employed to block the de novo RNA synthesis, followed by qRT-PCR analysis.

**Table 1.** Primers sequences used for qRT-PCR.

Name			Primers for qRT-PCR
circ_0092012		Forward	TGGCACTTACAGCTGCTCCT
		Reverse	CTCTACCGTGCCCTTCTGTC
PDL1		Forward	TTGCTGAACGCCCCATACAA
		Reverse	TCCAGATGACTTCGGCCTTG
miR-635		Forward	GCCGAGACTTGGGCACTGAAACA
		Reverse	GTGCAGGGTCCGAGGT
GAPDH		Forward	GACAGTCAGCCGCATCTTCT
		Reverse	GCGCCCAATACGACCAAATC
U6		Forward	CTCGCTTCGGCAGCACA
		Reverse	AACGCTTCACGAATTTGCGT
**Primers sequences used for RT-qPCR.**			
Name	Primers for RT-qPCR		
miR-635	GTCGTATCCAGTGCGTGTCGTGGAGTCGGCAATTGCACTGGATACGACGGACAT		

### Cell transfection

For transient transfection, 50 nM circ_0092012 small interfering RNA (siRNA) (si-circ_0092012), 50 ng pCD5-ciR/circ_0092012 overexpression vector (circ_0092012), 50 ng pcDNA 3.1/PDL1 overexpression vector (PDL1), 100 nM miR-635 mimic (miR-635) or inhibitor (anti-miR-635) or equal content negative control (si-NC, pCD5-ciR, pcDNA, miR-NC, or anti-miR-NC) (GeneChem, Shanghai, China) were co-transfected into H1299 and A549 cells.

For stable cell-line establishment, lentiviral plasmids pLCDH-ciR carrying shRNA targeting circ_0092012 or the nontarget short hairpin RNA (shRNA) (GeneChem), named as sh-circ_0092012 or sh-NC, were generated in 293 T cells. Then, viral supernatants were collected, and A549 cells were infected in complete medium containing 8 μg/mL polybrene. Finally, stably infected cells were selected by puromycin for animal experiments.

### Cell count kit-8 (CCK-8) assay

H1299 and A549 cells were placed at a 96-well plate and subjected to assigned transfection. 48 h later, each well was reacted with 10  μL CCK-8 solution (Beyotime, Beijing, China) for 2 h. The optical density value was examined at 450 nm by a microplate reader (Biotek, Winooski, Vermont).

### 5-ethynyl-2’-deoxyuridine (EdU) assay

EdU assay was carried out as per the protocols of EdU cell proliferation kit (Ribobio, Guangdong, China) [32]. After transfection, H1299 and A549 cells stained with EdU, Apollo, and 4’, 6-diamidino-2-phenylindole (DAPI) successively. Lastly, EdU positive cells were counted.

### Flow cytometry

Transfected H1299 and A549 cells were collected and stained with Annexin V-FITC/PI Apoptosis Detection Kit (BestBio, Shanghai, China) based on the manufacturer’s protocols [33]. The apoptotic cells were determined by a flow cytometry.

### Transwell assay

Following indicated transfection, H1299 and A549 cells in 200 μL serum-free medium were seeded into the upper chamber of transwell inserts with Matrigel-coated membrane (BD Biosciences). The lower chamber was filled with 500 μL of complete culture medium. 24 h later, invaded cells on the bottom of the lower compartment were fixed, stained, and counted.

### Wound healing assay

H1299 and A549 cells subjected to assigned transfections were seeded into a 6-well plate and scraped using a sterile pipette tip. At 0 and 24 h, Representative images were captured and cell migration was analyzed by Image J.

### Western blotting

Collected tissues and cells were homogenized with RIPA buffer and the concentration of protein was quantified by a BCA method. Then, protein extracts were loaded onto 10% SDS-PAGE for separating and transferred to a PVDF membrane. The membrane was incubated with primary antibodies at 4  C overnight, followed by interaction with secondary antibody at 37  C for 2 h. The antibodies used in this study included: Cleaved-caspase-3 (1:2000, ab2302), Caspase-3 (1:1000, ab13847), matrix metalloproteinase-2 (MMP2) (1:500, ab37150), PDL1 (1:1000, ab213480) and glyceraldehyde-3-phosphate dehydrogenase (GAPDH) (1:1000, ab181602), all provided by Abcam (Cambridge, UK). Finally, protein bands were visualized by an ECL kit (Beyotime) [34].

### Immune escape analysis

The peripheral blood was obtained from healthy individuals at Binhai County People’s Hospital. The CD8 + T cells were isolated as per the protocol of EasySep™ Direct Human CD8 + T cell Isolation Kit, and then co-cultured at 37  C for 24 h with targeted cells H1299 and A549 that were subjected to assigned transfection. Finally, the apoptosis of CD8 + T cells was detected using flow cytometry.

Peripheral blood mononuclear cells (PBMCs) were also obtained by density gradient centrifugation with Ficoll from peripheral blood as described previously [35]. Then, PBMCs were activated by phytohemagglutinin (PHA) treatment [36], followed by the co-culture with assigned H1299 and A549 cells. Later on, the levels of interferon-γ (IFN-γ) and tumor necrosis factor-α (TNF-α) released by PBMCs under PHA stimulation were detected by enzyme-linked immunosorbent assay (ELISA) with commercial kits (Abcam).

### Dual-luciferase reporter assay

The fragments of circ_0092012 and PDL1 3ʹuntranslated regions (3ʹUTRs) covering the miR-635 wild type (WT) binding sites and the mutated (MUT) sequences were amplified and inserted into the pmirGLO report luciferase vector (Invitrogen) to establish luciferase vectors. Next, these luciferase reporter plasmids together with miR-635 mimic or control mimic (miR-NC) were co-transfected into H1299 and A549 cells for 48 h, followed by luciferase activities detection with Dual-Lucy Assay Kit (Solarbio).

### RNA immunoprecipitation (RIP) assay

The lysates of H1299 and A549 cells were collected and reacted with magnetic beads (Millipore) and anti-Ago2 or negative IgG antibody overnight at 4  C. After being mixed with proteinase K for 30 min, levels of molecules were tested by qRT-PCR [16].

### Tumor xenograft assay

Stably infected A549 cells (2 × 10^5^ cells) were subcutaneously injected into BALB/c nude mice (N = 6/per group, four-weeks-old). After 8 days of inoculation, the size of xenografts was detected every 3 days and the volume was recorded following the volume = (length × width2)/2. Twenty-three days later, the xenograft tumors were isolated, weighed and divided either for IHC analysis as described previously [37]. This animal study was approved by the Animal Care Committee of Binhai County People’s Hospital.

### Statistical analysis

The results were manifested as mean (SD). Multiple comparisons were conducted by analysis of variance (ANOVA), and paired or unpaired *t*-test, or Mann–Whitney test was utilized for the comparisons of two-groups. *P* < 0.05 indicated significant differences. (*P < 0.05, **P < 0.01, ***P < 0.001, ****P < 0.0001)

## Results

In this study, we hypothesized that circ_0092012 might be involved in NSCLC tumorigenesis. Herein, this study aimed to investigate the role and molecular mechanisms of circ_0092012 on NSCLC malignant phenotype and immune escape. It was found that circ_0092012 was highly expressed in NSCLC tissues and cells. Circ_0092012 silencing suppressed NSCLC cell proliferative, migratory and invasive abilities and alleviated the immune suppressive effect on NSCLC cells. Mechanistically, circ_0092012 sponged miR-635, which targeted PDL1. Circ_0092012 exerted its effects via miR-635/PDL1 axis.

### Circ_0092012 is highly expressed in NSCLC and associated with the pathological stage

**Figure 1.**
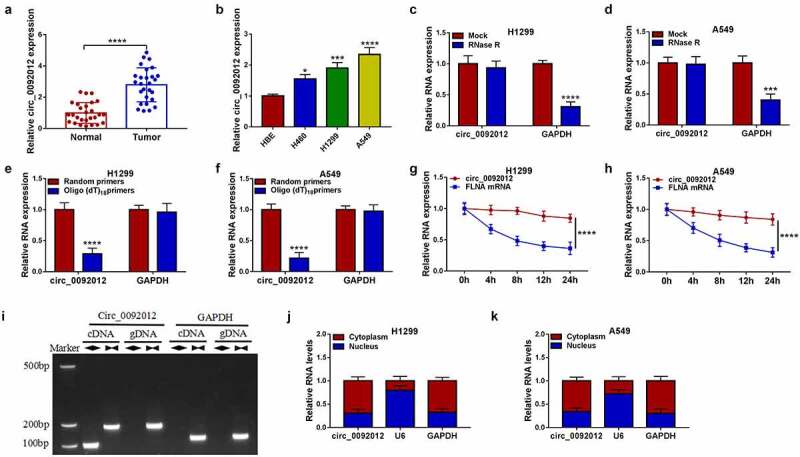
Circ_0092012 is highly expressed in NSCLC tissues and cells. (a, b) The expression of circ_0092012 in 27 pairs of NSCLC and adjacent normal tissues, as well as in NSCLC cells and HBE cells was detected using qRT-PCR. (c, d) qRT-PCR analysis of circ_0092012 expression in H1299 and A549 cells treated with RNase R or Mock. (e, f) qRT-PCR analysis of circ_0092012 in reverse transcription using Random and Oligo(dT)18 primers in H1299 and A549 cells. (g, h) qRT-PCR analysis of FLNA and circ_0092012 expression in H1299 and A549 cells after Actinomycin D treatment. (i) The linear GAPDH and circ_0092012 in cDNA and gDNA were amplified in H1299 and A549 cells by using convergent and divergent primers, respectively. (j, k) Nuclear-cytoplasmic fractionation assay was used to investigate the subcellular localization of circ_0092012 in H1299 and A549 cells. *P < 0.05.

Compared with normal tissues, circ_0092012 was highly expressed in NSCLC tissues (Figure 1(a)), and significantly correlated with TNM grade, lymph node metastasis and tumor size of NSCLC (Table 2). Similarly, we also observed an increase of circ_0092012 expression in NSCLC cell lines (Figure 1(b)). Thereafter, we found that circ_0092012 could resistant to the digestion by RNase R, a 3’ to 5’ exoribonuclease (Figure 1(c,d)). Moreover, Random and Oligo(dT)18 primers were used in reverse transcription experiments, the results showed that circ_0092012 expression was decreased relative to the linear GAPDH transcript in H1299 and A549 cells (Figure 1(e,f)). Furthermore, we used actinomycin D to inhibit transcription and then the results showed that the half-life of circ_0092012 exceeded 24 h, while that of linear FLNA was about 4 h in H1299 and A549 cells (Figure 1(g,h)). Meanwhile, the convergent primers and divergent primers were used to amplify linear GAPDH and circ_0092012 using cDNA and genomic DNA (gDNA) as templates, it was observed that circ_0092012 amplification products were only detected in cDNA by divergent primers but not in gDNA (Figure 1(i)), suggesting that circ_0092012 could be formatted. In addition, circ_0092012 level was higher in cytoplasmic fraction than the nuclear fraction (Figure 1(j,k)).

**Table 2.** Relationship between circ_0092012 expression and clinicopathologic features of NSCLC patients.

		circ_0092012 expression		
	Characteristics n = 27	Low(n = 13)	High(n = 14)	*P* value^a^
Gender				0.7036
Female	11	6	5	
Male	16	7	9	
Age (years)				0.6946
≤60	9	5	4	
>60	18	8	10	
TNM grade				0.0213*
I+ II	12	9	3	
III+IV	15	4	11	
Lymph node metastasis				0.0183*
Positive	17	5	12	
Negative	10	8	2	
Tumor size				0.0461*
≤3 cm	9	7	2	
>3 cm	18	6	12	

TNM, tumor-node-metastasis; *P < 0.05 ^a^Chi-square test

### *Circ_0092012 silencing suppresses NSCLC cell malignant biological behaviors* in vitro

**Figure 2.**
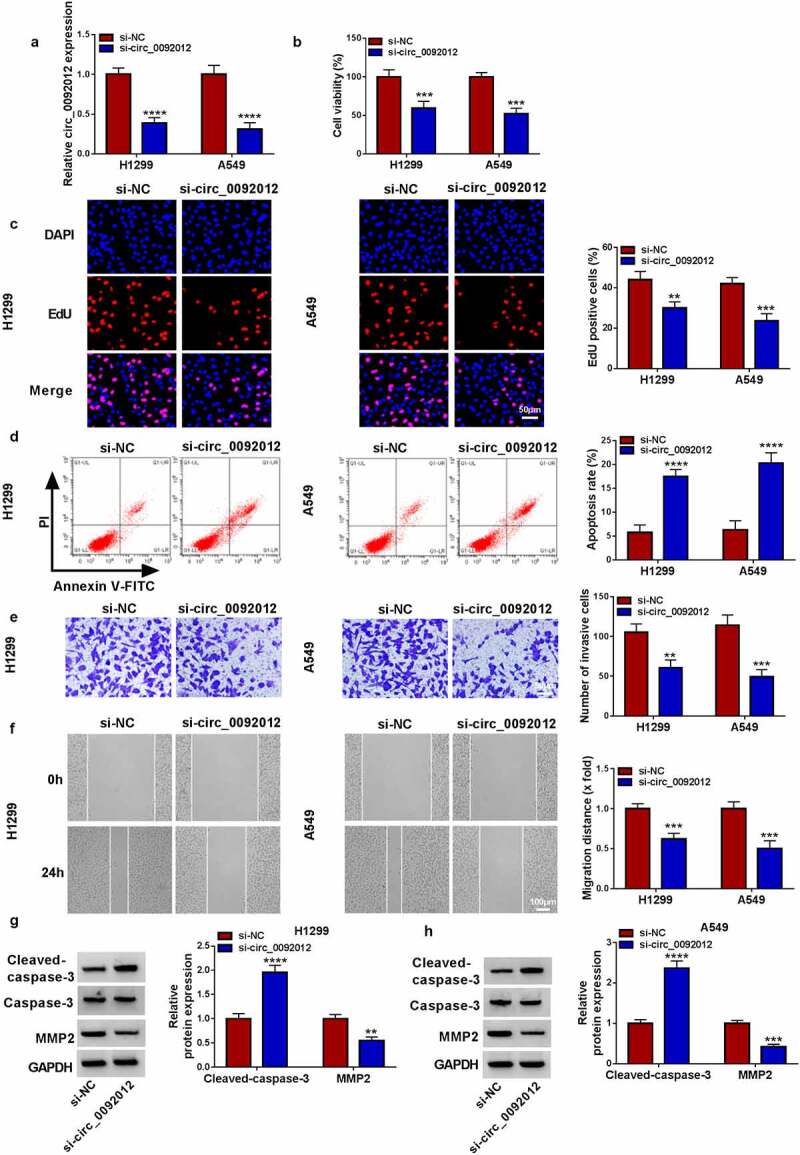
Circ_0092012 silencing suppresses NSCLC cell malignant biological behaviors *in vitro*. (a) The knockdown efficiency of si-circ_0092012 and si-NC in H1299 and A549 cells was validated using qRT-PCR. The proliferation (b, c), apoptosis (d), invasion (e) and migration (f) of H1299 and A549 cells transfected with si-circ_0092012 and si-NC were determined. (g, h) Western blotting for caspase-3, cleaved-caspase-3 and MMP2 protein levels. *P < 0.05.

To determine the biological functions of circ_0092012 in NSCLC, we established circ_0092012 siRNA to conduct loss-of-function experiments. The transfection of si-circ_0092012 significantly reduced circ_0092012 expression level, but not affected the parental gene FLNA (Figure 2(a) and Fig. S1). Functionally, circ_0092012 deficiency reduced cell proliferation in H1299 and A549 cells (Figure 2(b,c)). Conversely, circ_0092012 knockdown led to an enhancement of the apoptosis in H1299 and A549 cells (Figure 2(d)). Thereafter, transwell and would healing assays indicated that circ_0092012 knockdown inhibited the invasive and migratory capabilities of H1299 and A549 cells (Figure 2(e,f)). MMP2 is a member of the zinc-dependent metalloproteinase gene family that can degrade most components of the extracellular matrix and basement membrane, thus playing an important role in modulating cancer invasion and metastasis [38–40]. As expected, western blotting analysis showed that circ_0092012 silencing reduced the level of MMP2, but elevated the level of Cleaved-caspase-3, a reliable marker for cells that are dying or have died by apoptosis [41], in H1299 and A549 cells (Figure 2(g,h)).

### Circ_0092012 knockdown inhibits immune escape in NSCLC cells

**Figure 3.**
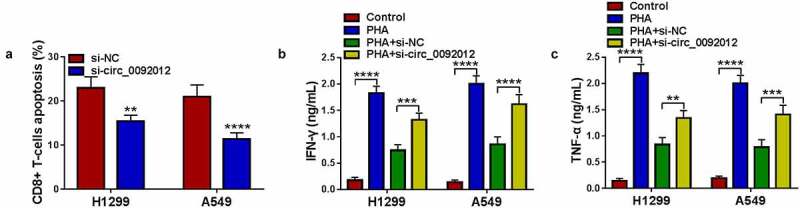
Circ_0092012 knockdown inhibits immune escape in NSCLC cells. (a) The apoptosis of CD8 + T cells was examined by flow cytometry. (b, c) Levels of IFN-γ and TNF-α were examined using ELISA. *P < 0.05.

Subsequently, we evaluated if circ_0092012 affected immune response in NSCLC cells. CD8 + T cells (effector cells) were co-cultured with H1299 and A549 transfected with si-circ_0092012 to determine the cytotoxicity of CD8 + T cells. The results showed that the apoptosis ratio of CD8 + T cells was decreased after co-cultured with circ_0092012-decreased H1299 and A549 cells relative to the negative control (Figure 3(a)). Moreover, the co-culture of CD8 + T cells with circ_0092012-decreased H1299 and A549 cells significantly suppressed the viability of H1299 and A549 cells, indicating the restoration of anti-tumor cytotoxicity of CD8 + T cells (Fig. S2). Besides that, the levels of IFN-γ and TNF-α in PBMCs stimulated by PHA were decreased when co-cultured with H1299 and A549 cells, which were rescued by circ_0092012 knockdown in cells (Figure 3(b,c)).

In addition, the effects of circ_0092012 overexpression in NSCLC cells were further investigated. The circ_0092012 overexpression plasmids were established, which transfection was found to significantly up-regulate circ_0092012 expression (Fig. S3A). Functionally, circ_0092012 up-regulation enhanced cell proliferation, invasion and migration in cell proliferation (Fig. S3B-E). Furthermore, circ_0092012 overexpression promote the immune suppressive effects, evidenced by increased CD8 + T cells apoptosis, as well as decreased release of IFN-γ and TNF-α in PHA-stimulated PBMCs (Fig. S3F-H).

### Circ_0092012 functions as a sponge for miR-635

**Figure 4.**
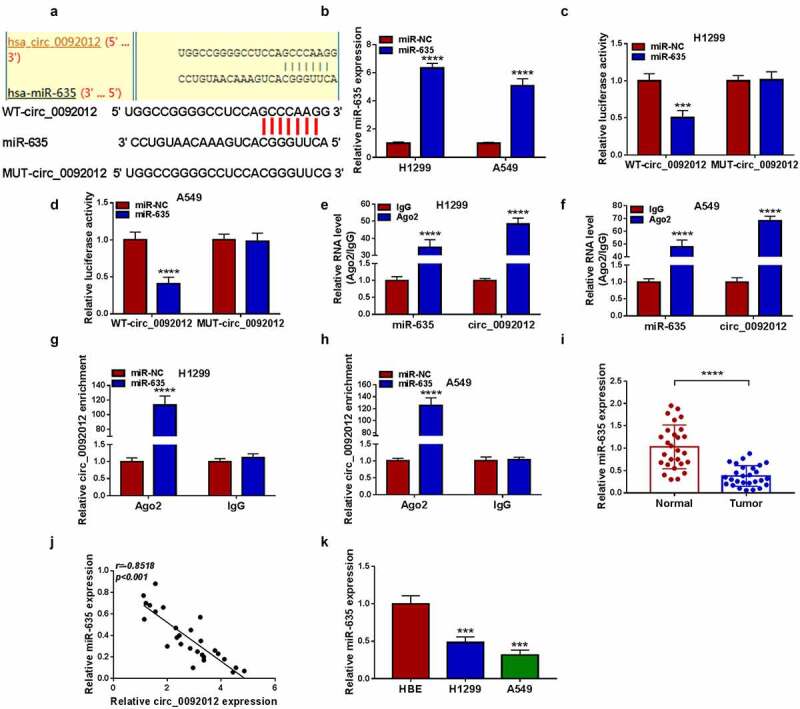
Circ_0092012 functions as a sponge for miR-635. (a) The predicted binding sequence of circ_0092012 and miR-635. (b) qRT-PCR for the analysis of the transfection efficiency of miR-635 or miR-NC. (c-h) The binding between circ_0092012 and miR-635 was validated using the dual-luciferase reporter assay and RIP assay. (i) Levels of miR-635 in 27 pairs of NSCLC patients was detected using qRT-PCR. (j) Negative correlation between circ_0092012 and miR-635 expression. (k) MiR-635 expression detection in NSCLC cells and HBE cells by qRT-PCR. *P < 0.05.

Whereafter, we predicted the potential miRNAs binding with circ_0092012 using circinteractome database. The results showed that miR-635 had multiple potential binding sites on circ_0092012 (Figure 4(a)). The transfection efficiency of miR-635 mimic was validated by qRT-PCR (Figure 4(b)). Then, we found miR-635 mimic overtly reduced the luciferase activity in WT-circ_0092012 group rather than MUT-circ_0092012 (Figure 4(c,d)). Meanwhile, circ_0092012 and miR-635 were enriched in AGO2 immunoprecipitates in comparison to the IgG antibody (Figure 4(e,f)), besides, both circ_0092012 and miR-635 were significantly enriched in cells transfected with miR-635 mimics compared with miR-NC group (Figure 4(g,h)). Thereafter, miR-635 expression was observed to be decreased in NSCLC tissues (Figure 4(i)), and inversely correlated with circ_0092012 expression (Figure 4(j)). Moreover, a decreased miR-635 expression was also observed in NSCLC cell lines (Figure 4(k)).

### Circ_0092012 silencing suppresses NSCLC cell oncogenic phenotypes and immune escape by targeting miR-635

**Figure 5.**
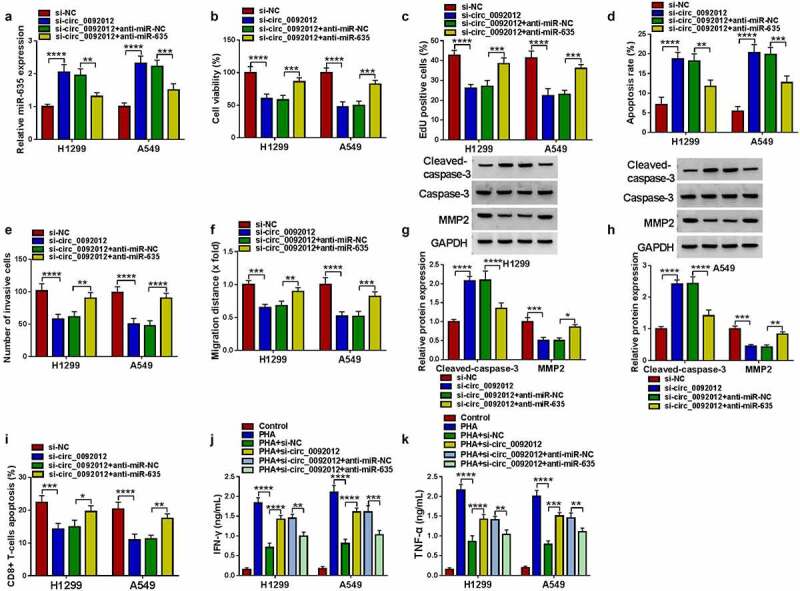
Circ_0092012 silencing suppresses NSCLC cell oncogenic phenotypes and immune escape by targeting miR-635. (a-k) H1299 and A549 cells were co-transfected with circ_0092012 siRNA and/or miR-635 inhibitor. (a) qRT-PCR analysis of miR-635 expression in H1299 and A549 cells. The proliferation (b, c), apoptosis (d), invasion (e) and migration (f) of H1299 and A549 cells were determined. (g, h) Western blotting for the caspase-3, MMP2 and cleaved-caspase-3 protein levels in cells. (i) The apoptosis of CD8 + T cells was examined by flow cytometry. (j, k) ELISA for the levels of IFN-γ and TNF-α. *P < 0.05.

Thereafter, whether miR-635 mediated the effects of circ_0092012 on NSCLC progression was further explored. The elevation of miR-635 expression caused by circ_0092012 silencing was reverted by anti-miR-635 transfection (Figure 5(a)). Then, miR-635 inhibition reversed circ_0092012 deficiency-induced inhibition of cell proliferation (Figure 5(b,c)), promotion of cell apoptosis (Figure 5(d)), and reduction of the number of invaded and migrated cells (Figure 5(e,f)) in H1299 and A549 cells. Meanwhile, miR-635 inhibitor decreased Cleaved-caspase-3 level and increased MMP2 level in circ_0092012-decreased H1299 and A549 cells (Figure 5(g,h)). Besides that, miR-635 inhibitor reversed circ_0092012-evoked inhibition of immune escape in H1299 and A549 cells (Figure 5(i-k)).

### PDL1 is targeted by miR-635

**Figure 6.**
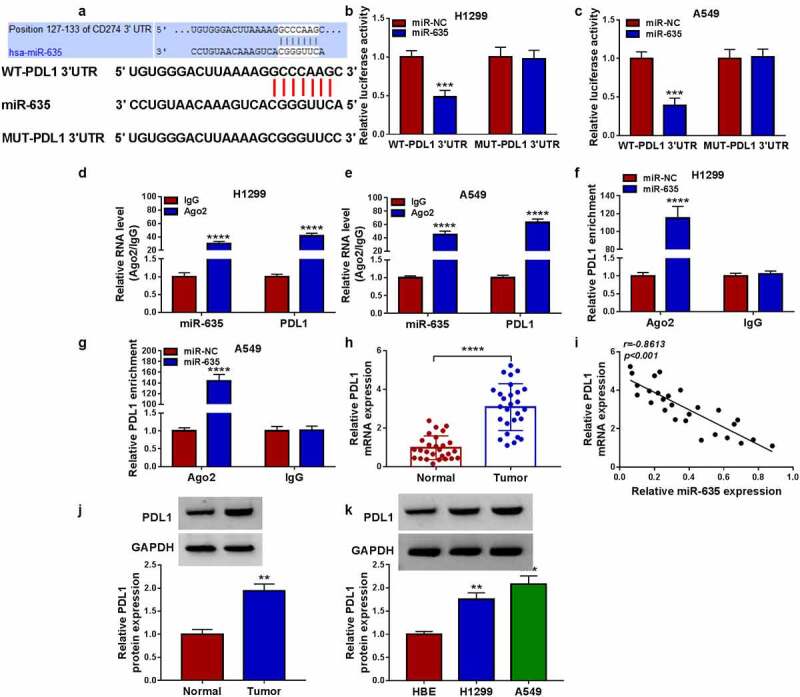
PDL1 is targeted by miR-635. (a) The complementary binding sequence of PDL1 and miR-635. (b-g) The binding between PDL1 and miR-635 was validated by the dual-luciferase reporter and RIP assays. (h) The expression of PDL1 mRNA in 27 pairs of NSCLC samples was tested using qRT-PCR. (i) Negative correlation between PDL1 and miR-635 expression. (j, k) Western blotting of PDL1 protein level in 27 pairs of NSCLC samples as well as in NSCLC cells and HBE cells. *P < 0.05.

The potential targets of miR-635 were predicted by Targetscan database, and we found that miR-635 had binding sites on PDL1 3’-UTR (Figure 6(a)). Then, the decline of the luciferase activity induced by miR-635 overexpression in H1299 and A549 cells transfected with wild-type PDL1 reporter vector was observed (Figure 6(b,c)). Moreover, the binding between them was also verified by RIP assay with high enrichment of PDL1 and miR-635 in anti-Ago2 group (Figure 6(d,e)), as well as the abundances of both PDL1 and miR-635 in cells transfected with miR-635 mimics (figure 6(f,g)). Besides that, miR-635 inhibitor led to an increase of PDL1 expression level in H1299 and A549 cells (Fig. S4). Thereafter, we found that PDL1 mRNA was increased in NSCLC tissues (Figure 6(h)) and was inversely correlated with miR-635 expression (Figure 6(t)). And the same increase was also observed in the protein level of PDL1 in NSCLC tissues (Figure 6(j)). Additionally, PDL1 expression was also elevated in NSCLC cells (Figure 6(k)).

### MiR-635 suppresses NSCLC cell malignant biological behaviors and immune escape by targeting PDL1

**Figure 7.**
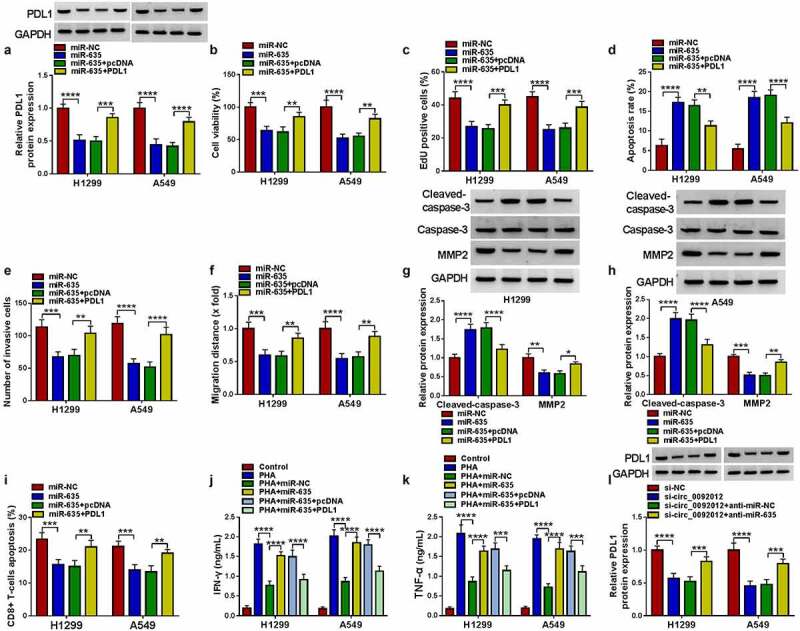
MiR-635 suppresses NSCLC cell malignant biological behaviors and immune escape by targeting PDL1. (a-k) H1299 and A549 cells were co-transfected with miR-635 mimic and/or PDL1 vector. (a) Western blotting of PDL1 expression in H1299 and A549 cells. The proliferation (b, c), apoptosis (d), invasion (e) and migration (f) of H1299 and A549 cells were determined. (g, h) Detection of the caspase-3, MMP2 and cleaved-caspase-3 protein levels in cells via Western blotting. (i) Flow cytometry for the apoptosis of CD8 + T cells. (j, k) Levels of IFN-γ and TNF-α were assayed using ELISA. (l) The level of PDL1 was detected in H1299 and A549 cells co-transfected with circ_0092012 siRNA and/or miR-635 inhibitor using western blotting. *P < 0.05.

The western blotting analysis showed that miR-635 mimic reduced the level of PDL1, which was reverted by PDL1 transfection in H1299 and A549 cells (Figure 7(a)). Thereafter, we proved that miR-635 overexpression suppressed cell proliferation (Figure 7(b,c)), promoted cell apoptosis (Figure 7(d)), and inhibited cell invasion and migration abilities (Figure 7(e,f)), and these effects were attenuated by PDL1 overexpression. Western blotting analysis manifested that PDL1 overexpression reversed miR-635 mimic-triggered increase of Cleaved-caspase-3 and decrease of MMP2 (Figure 7(g,h)). In addition, H1299 and A549 cells transfected with miR-635 mimic were sufficient to suppress the apoptosis of CD8 + T cells, while increase of PDL1 in miR-635-overexpressed cells attenuated these effects (Figure 7(i)). Furthermore, the levels of IFN-γ and TNF-α released by PHA-stimulated PBMCs were partially elevated by co-culturing with miR-635 increased H1299 and A549 cells, while these effects were counteracted by subsequent PDL1 overexpression in miR-635-overexpressed cells (Figure 7(j,k)).

Additionally, western blotting analysis showed that circ_0092012 deficiency resulted in a decrease of PDL1 expression, which was rescued by the inhibition of miR-635 (Figure 7(l)). Besides that, western blotting analysis showed that circ_0092012 overexpression reversed miR-635-caused decrease of PDL1 in H1299 and A549 cells (Fig. S5), indicating the circ_0092012/miR-635/PDL1 axis in NSCLC cells

### *Circ_0092012 deletion impedes NSCLC growth* in vivo

**Figure 8.**
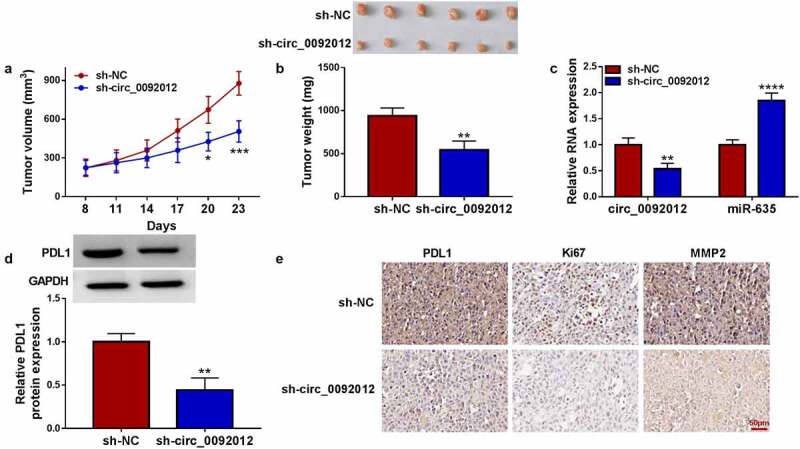
Circ_0092012 knockdown impedes NSCLC tumor growth *in vivo*. (a, b) The *in vivo* growth curve, representative images (upper panel), and tumor weight of xenografts. (c, d) Levels of circ_0092012, miR-635 and PDL1 in xenografts. (e) IHC staining for the protein of PDL1, Ki67 and MMP2 in the subcutaneous xenografts. *P < 0.05.

To evaluate the tumor-promoting effects of circ_0092012 *in vivo*, the animal experiment was conducted. Compared with si-NC group, deletion of circ_0092012 reduced the volume and weight of subcutaneous xenografts (Figure 8(a,b)). The intratumoral circ_0092012 and PDL1 expression were decreased, while miR-635 level was increased in xenografts of sh-circ_0092012 group (Figure 8(c,d)). Additionally, IHC staining showed the decreased PDL1, Ki67 and MMP2 protein levels in xenografts of sh-circ_0092012 group (Figure 8(e)).

## Discussion

Currently, accumulating evidence suggests that circRNAs are altered in NSCLC patients and tightly associated with the pathophysiological process of this cancer. For example, hsa_circRNA_101237 was found to be increased in NSCLC, and predicted poor survival, besides that, the up-regulation of hsa_circRNA_101237 promoted cell growth and metastasis [42]. Hong *et al*. demonstrated that circ-CPA4 promoted NSCLC cell growth, stemness, immune escape and enhanced drug resistance [43]. CircP4HB was showed to sponge miR-133a-5p and then accelerated cell metastasis in NSCLC [44]. Thus, circRNAs might be potential therapeutic biomarkers for NSCLC. In our work, an increase of circ_0092012 expression in NSCLC was found. Functionally, knockdown of circ_0092012 suppressed NSCLC tumorigenesis *in vitro* and in subcutaneous xenograft model, suggesting that circ_0092012 siRNA had tumor-suppressive effects on NSCLC progression.

Given the ceRNA hypothesis [18], the miRNA/mRNA axis underlying circ_0092012 was further investigated. The results identified the circ_0092012/miR-635/PDL1 axis in NSCLC cells. Previous studies have suggested the anticancer effects of miR-635 in NSCLC tumorigenic ability by regulating cell malignant biological behaviors and glycolysis [25,45,46]. In this study, we also showed the same anticancer action of miR-635 in NSCLC cell growth and mobility. Importantly, we found that miR-635 inhibition reversed the tumor-suppressive functions mediated by circ_0092012 siRNA in NSCLC cells. Immune system plays important roles in eliminating cancer cells. It has been discovered that PDL1 expressed on tumor cells can inhibit antitumor function of T cells by protecting cancer cells from immune responses through PD-1/PDL1 pathway [47,48]. Additionally, NSCLC cells have been identified that could escape the surveillance of immune system by interacting with immune checkpoint molecules expressed on regulatory T cells (e.g. PD-1) and subsequent inhibition of the activation of regulatory T cells [49]. Currently, immune inhibitors targeting the PD-1/PDL1 axis have been reported to be effective anti-cancer therapy to improve the clinical outcome of advanced NSCLC [50,51]. In current work, an amplified abundance of PDL1 was proved in NSCLC, moreover, PDL1 up-regulation abated the anticancer action of miR-635 in NSCLC. Additionally, given the role of PDL1 in immune escape, we investigated the action of circ_0092012/miR-635/PDL1 axis in immune suppressive effect in NSCLC cells. The results confirmed that circ_0092012 could inhibit the function of CD8 + T cells and reduce the levels of IFN-γ and TNF-α released by PHA-stimulated PBMCs through miR-635/PDL1 axis.

## Conclusion

In all, we firstly demonstrated that circ_0092012 knockdown could suppress tumor-autonomous malignant phenotypes and immune escape by miR-635/PDL1 axis in NSCLC, which provided new insights into the action pattern of circ_0092012 in NSCLC tumorigenesis and progression and the promising therapeutic targets for the development of RNA-based therapy in NSCLC patients.

## Supplementary Material

Supplemental Material
