# Supplementary material for: Circ_0092012 knockdown restrains non-small cell lung cancer progression by inhibiting cell malignant phenotype and immune escape through microRNA-635/programmed death ligand 1 axis
Source: Bioengineered. 2022 Jun 19;13(5):13929–43. doi: 10.1080/21655979.2022.2080386 (PMC9276036; doi:10.1080/21655979.2022.2080386)

The original western blots of Fig2

G

Repeat 1 | Repeat 2 | Repeat 3

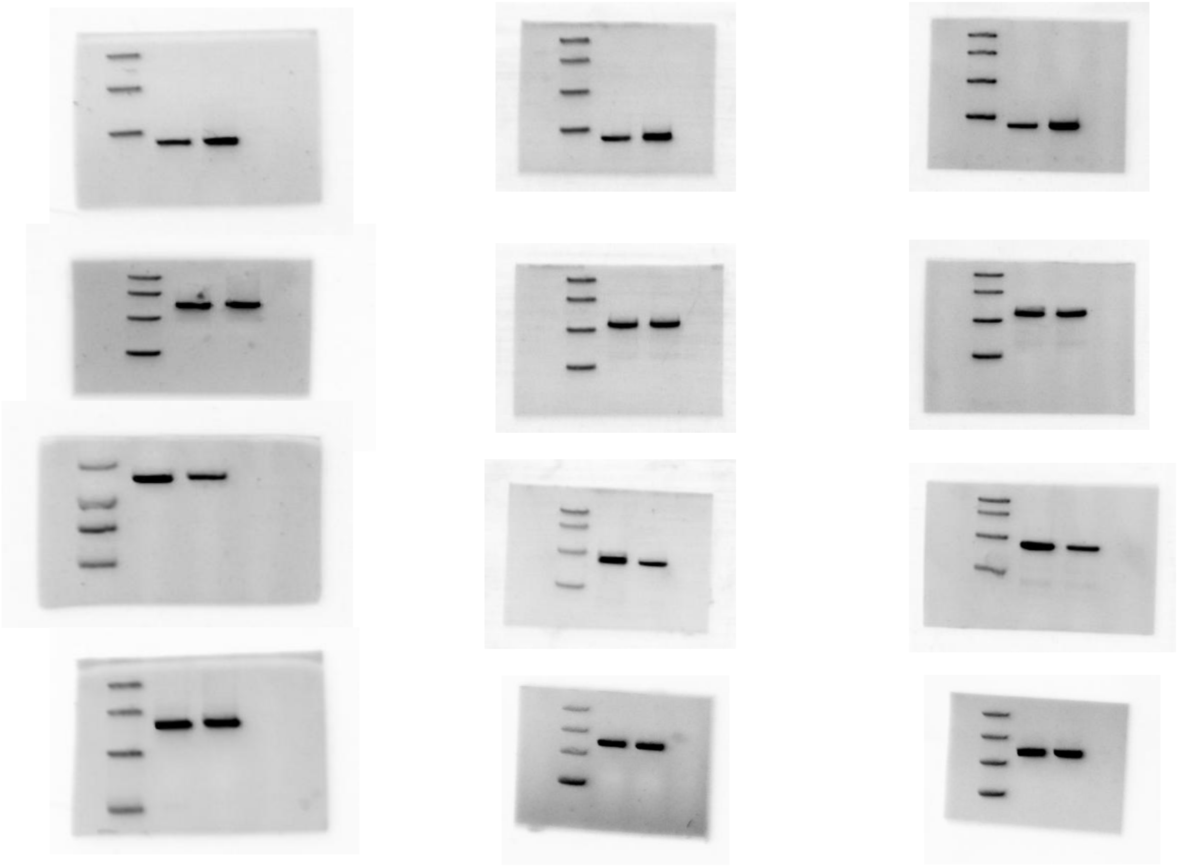

Cleaved-caspase-3

Caspase-3

MMP2

GAPDH

The original western blots of Fig2

H

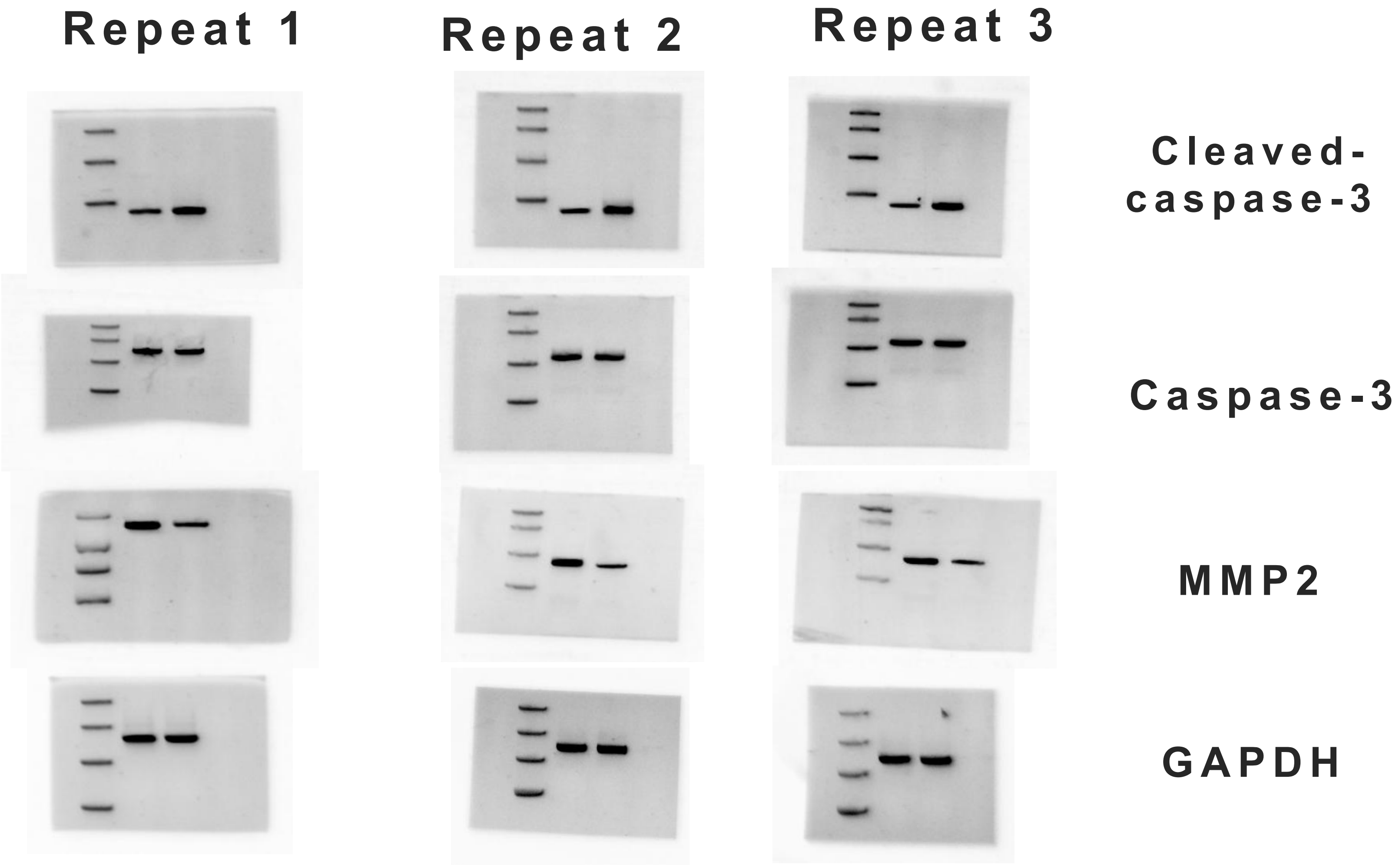

The original western blots of Fig5

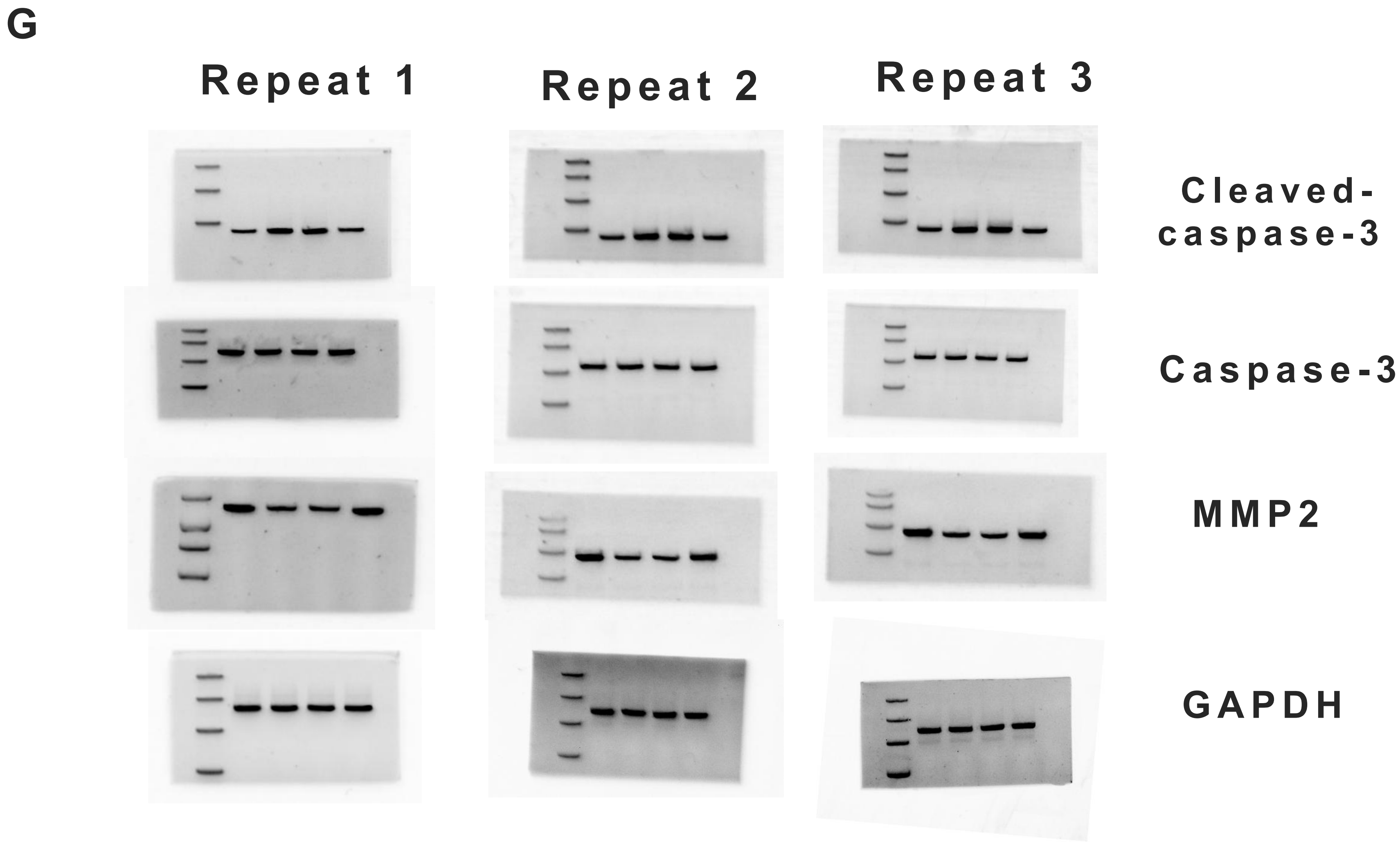

The original western blots of Fig5

H

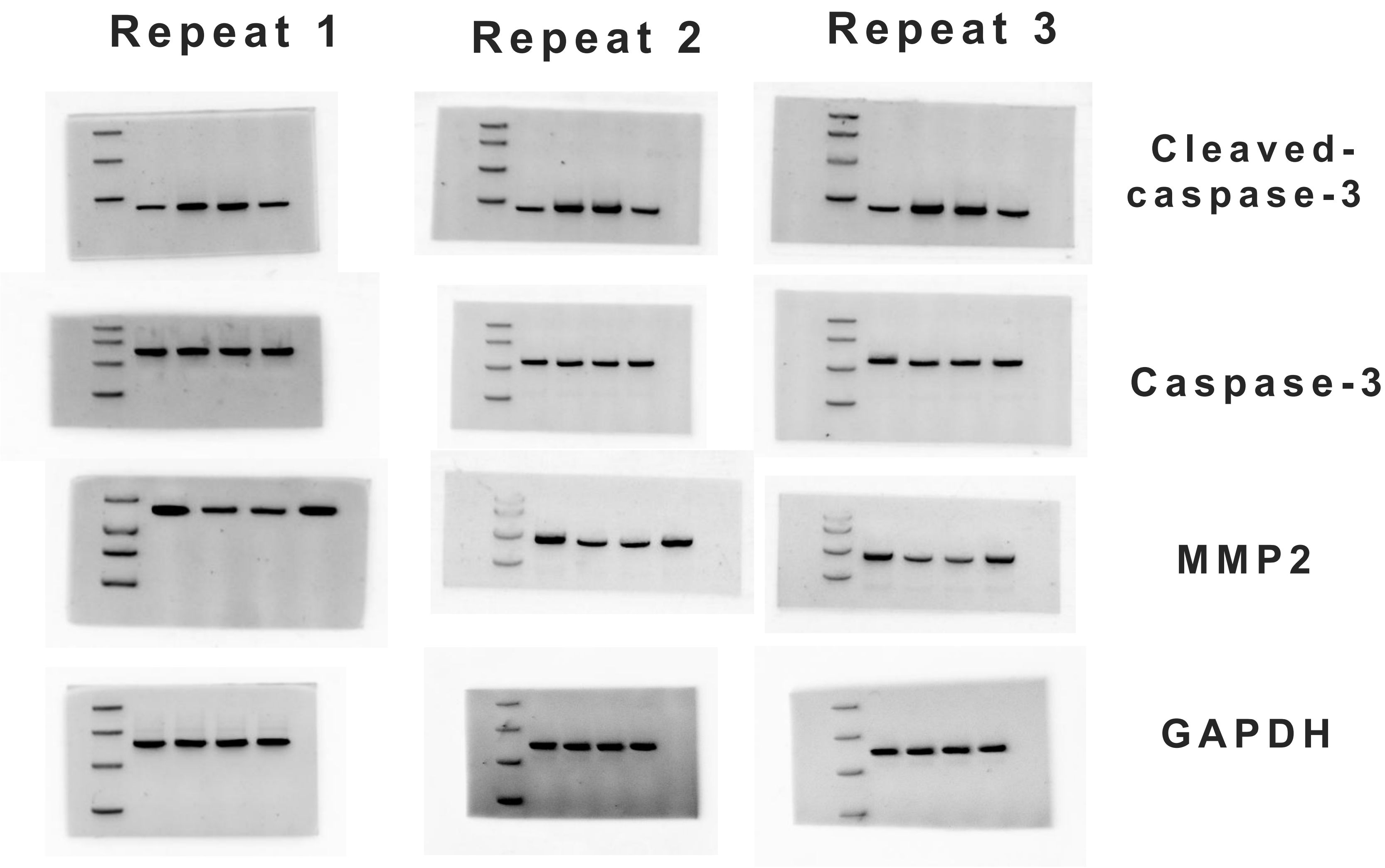

The original western blots of Fig6

H

Repeat 1 **Repeat 2** **Repeat 3**

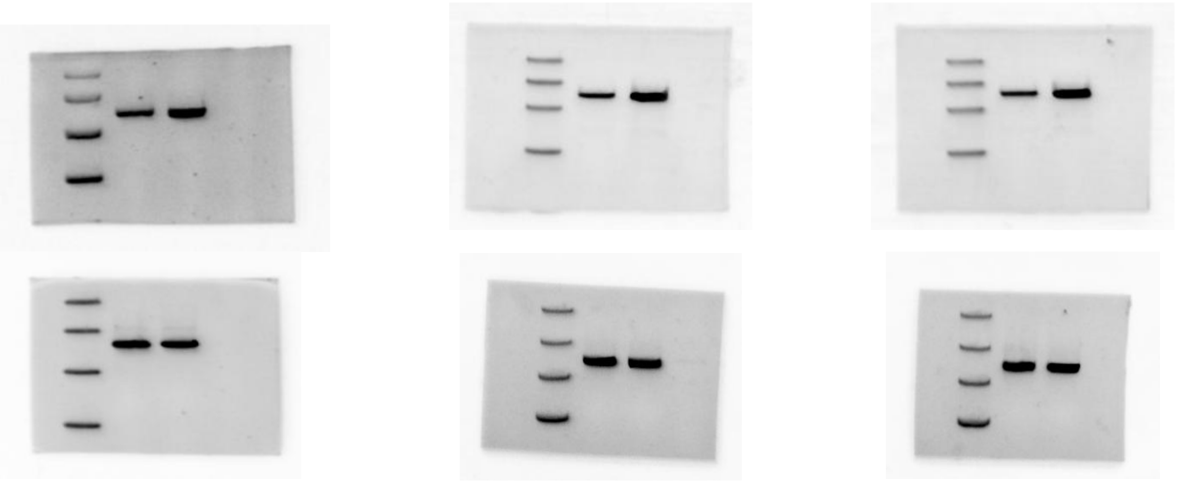

PDL1

GAPDH

The original western blots of Fig6

I

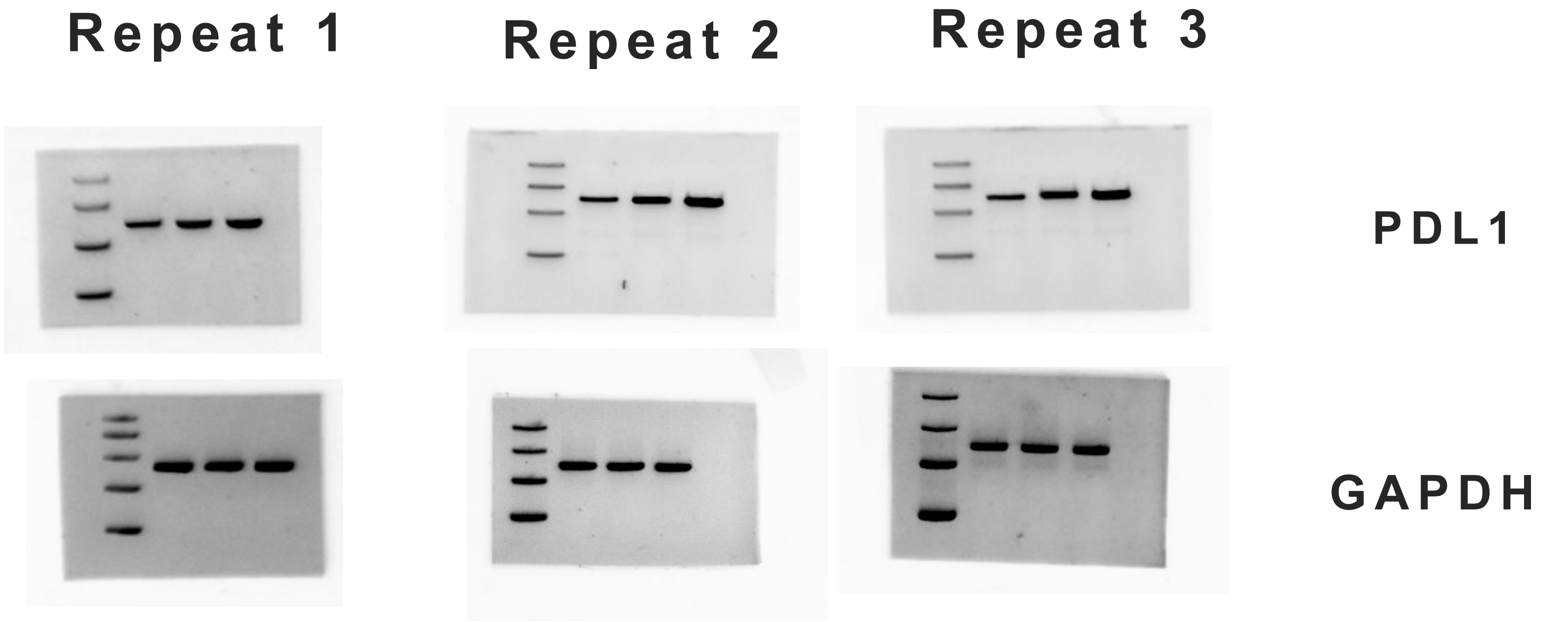

The original western blots of Fig7

A

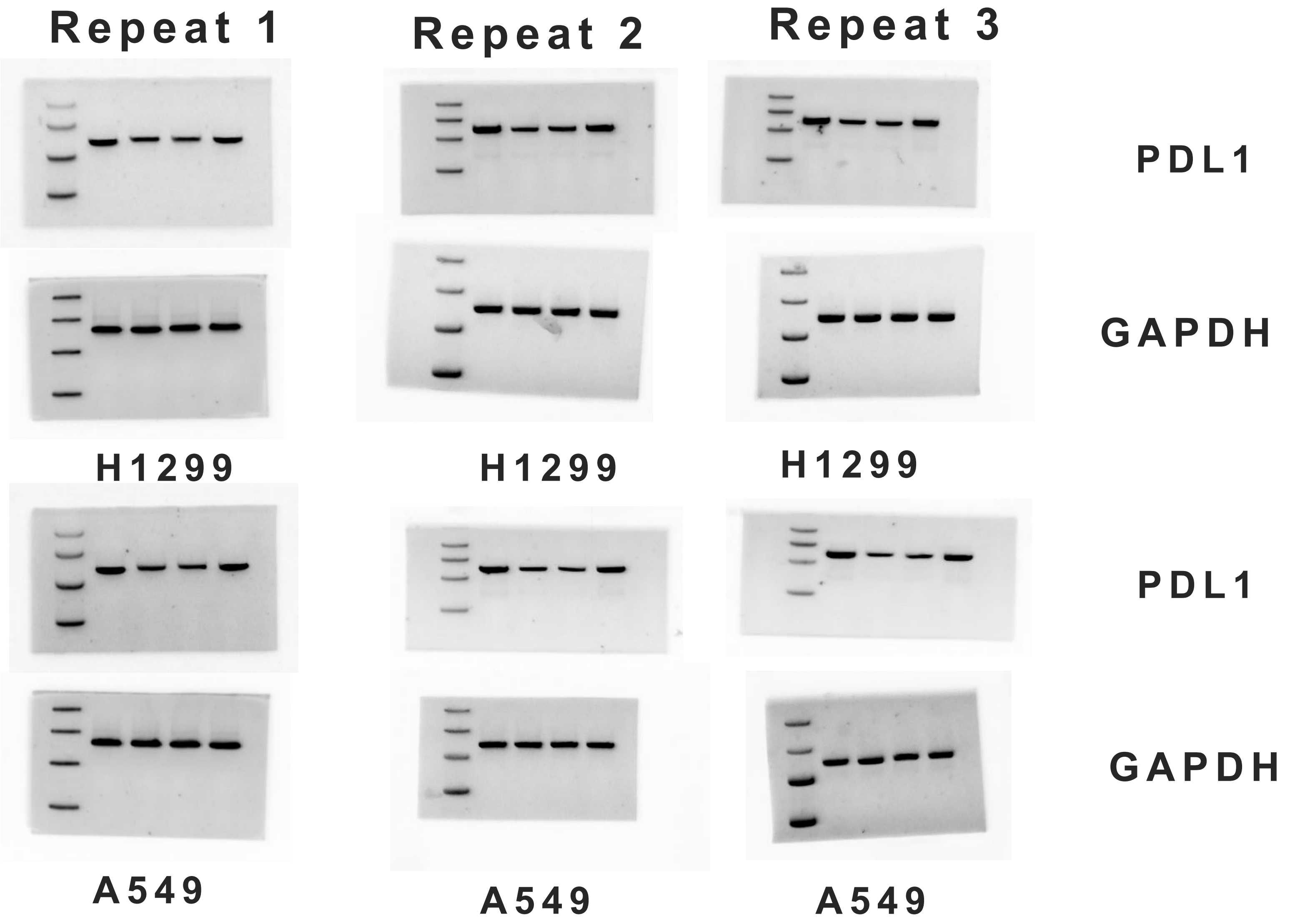

The original western blots of Fig7

G

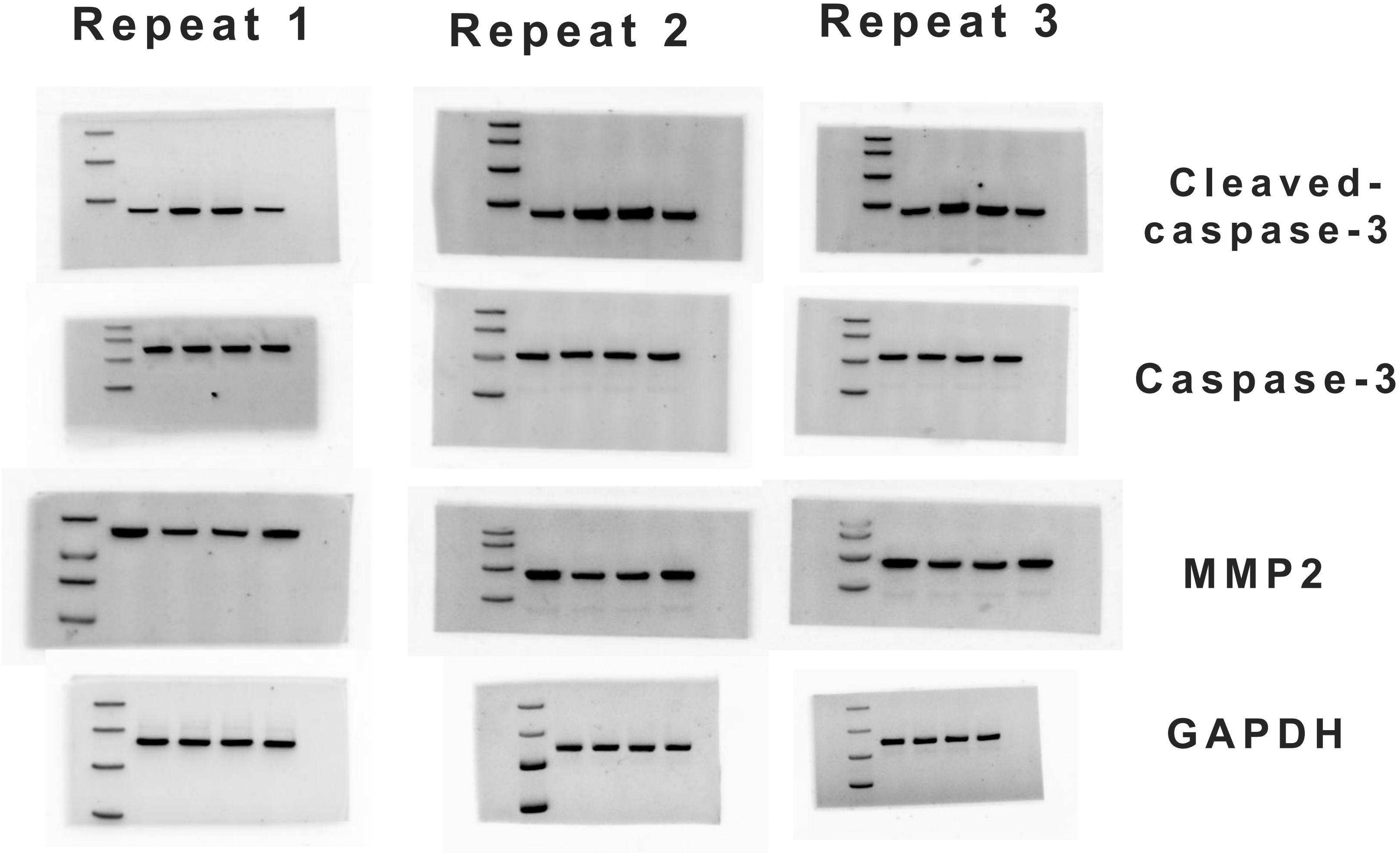

The original western blots of Fig7

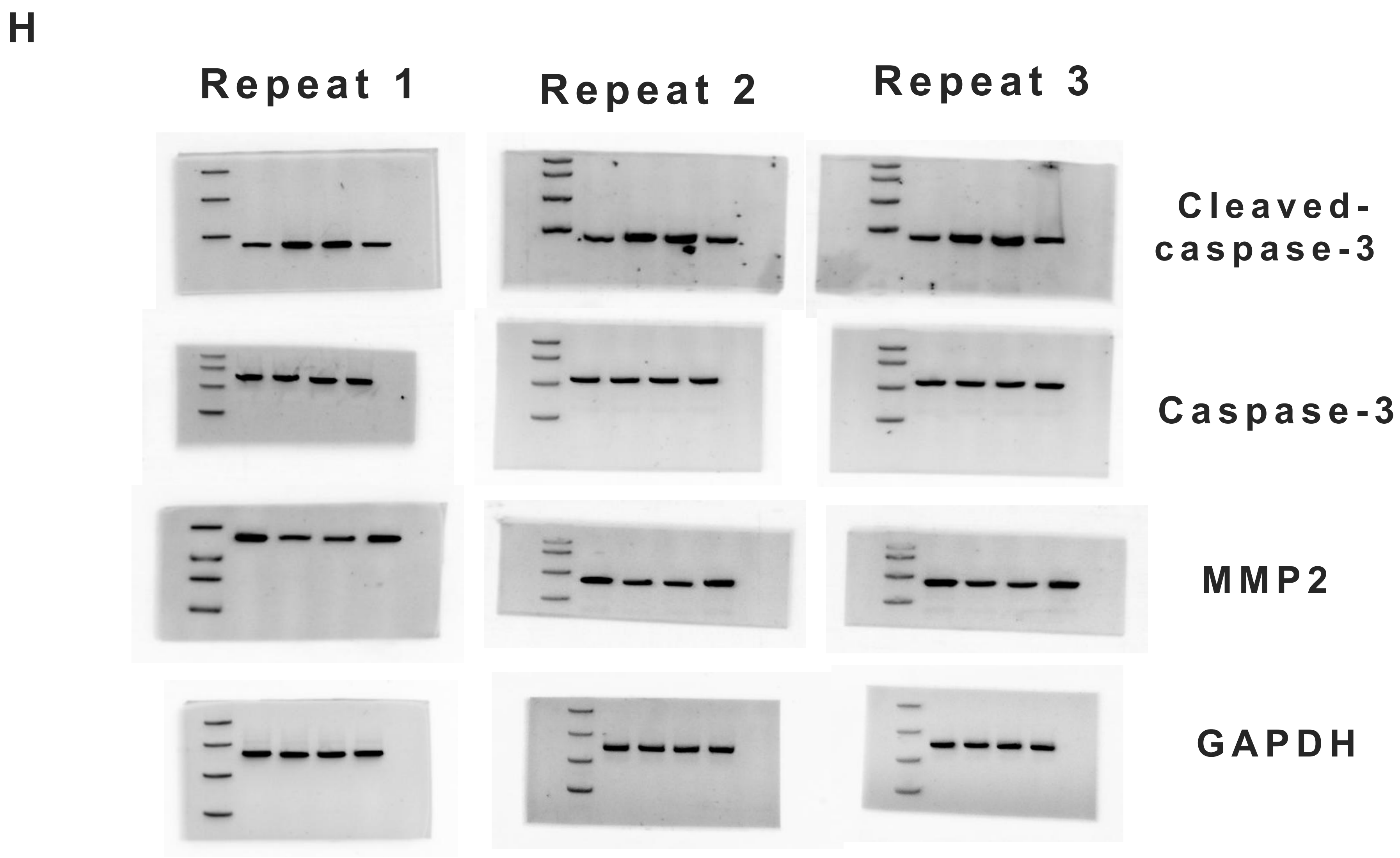

The original western blots of Fig7

L

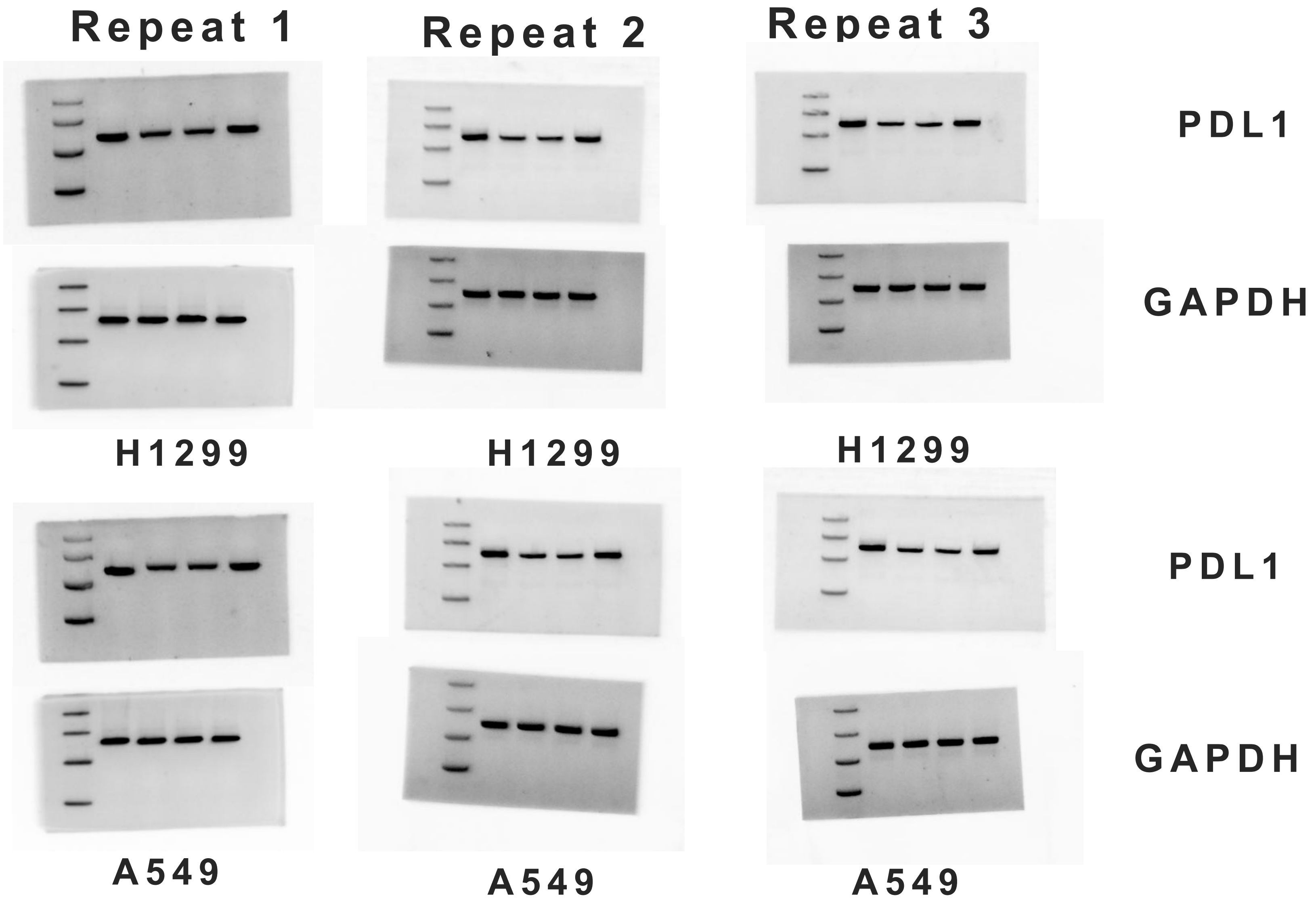

The original western blots of Fig8

D

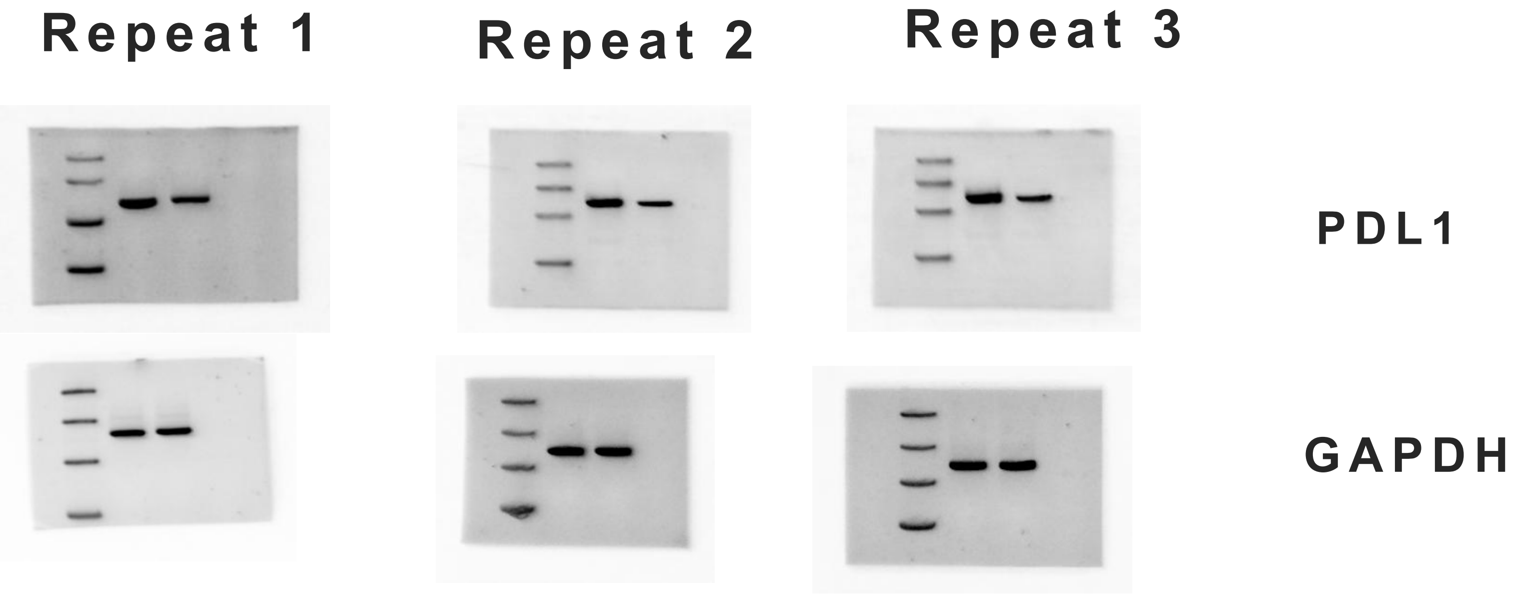

Supplement: Supplemental Material [file KBIE_A_2080386_SM5074.zip › original images of western blots.pdf]
