# Supplementary figures and images for: Circ_0092012 knockdown restrains non-small cell lung cancer progression by inhibiting cell malignant phenotype and immune escape through microRNA-635/programmed death ligand 1 axis
Source: Bioengineered. 2022 Jun 19;13(5):13929–43. doi: 10.1080/21655979.2022.2080386 (PMC9276036; doi:10.1080/21655979.2022.2080386)

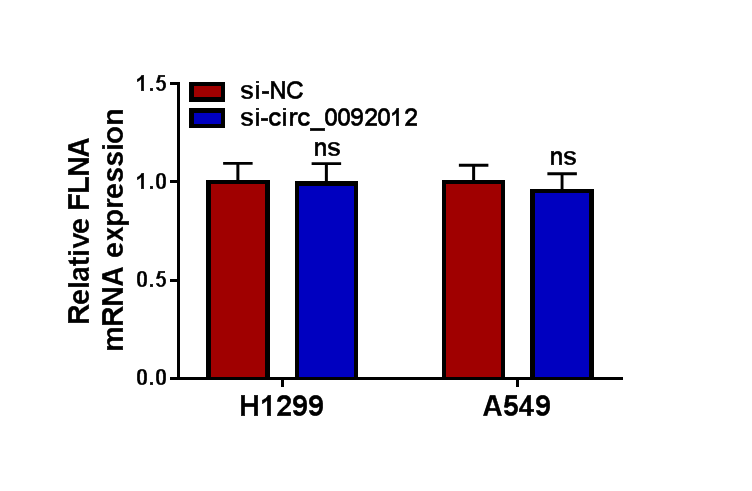

Supplement: Supplemental Material [file KBIE_A_2080386_SM5074.zip › Figure S1.tif]

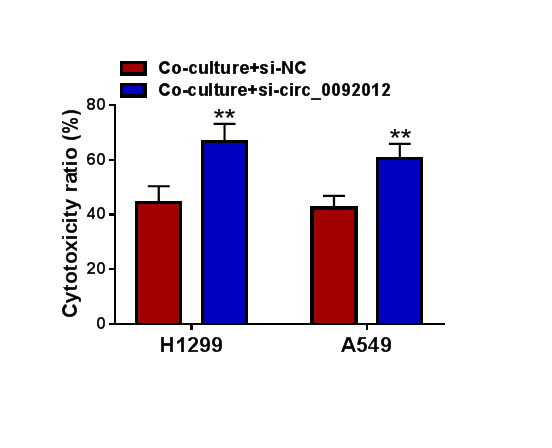

Supplement: Supplemental Material [file KBIE_A_2080386_SM5074.zip › Figure S2.tif]

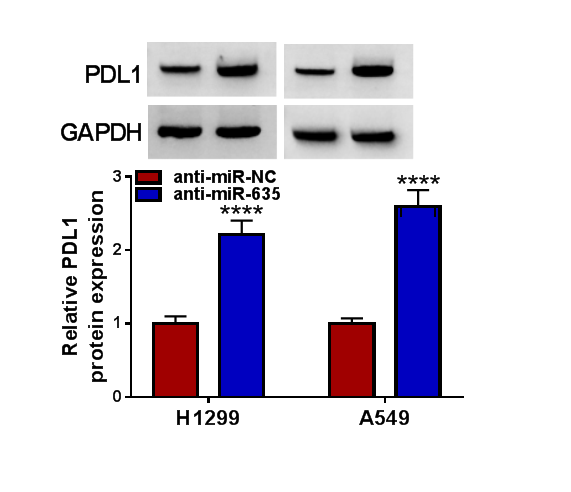

Supplement: Supplemental Material [file KBIE_A_2080386_SM5074.zip › Figure S4.tif]

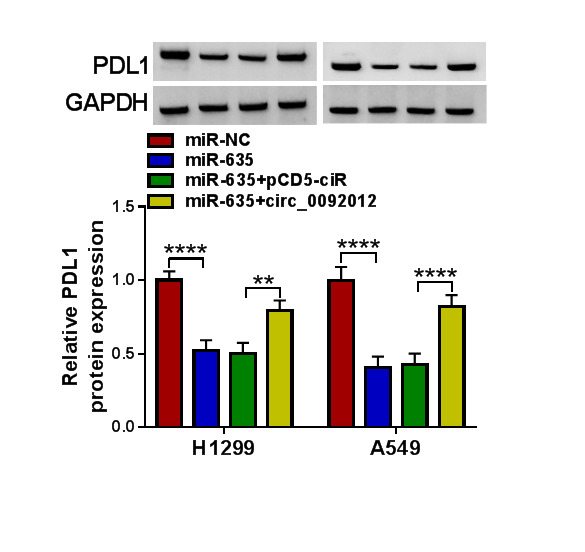

Supplement: Supplemental Material [file KBIE_A_2080386_SM5074.zip › Figure S5.tif]

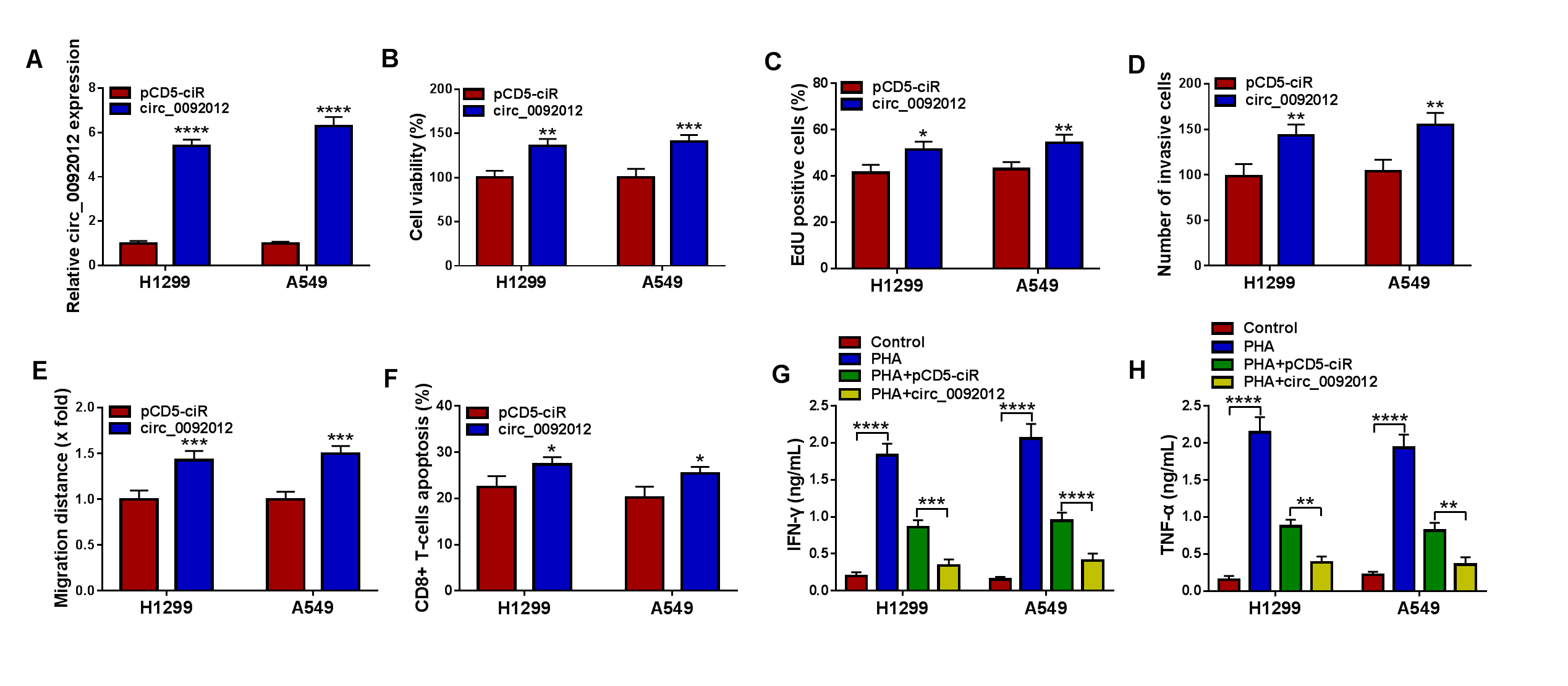

Supplement: Supplemental Material [file KBIE_A_2080386_SM5074.zip › FigureS3.tif]

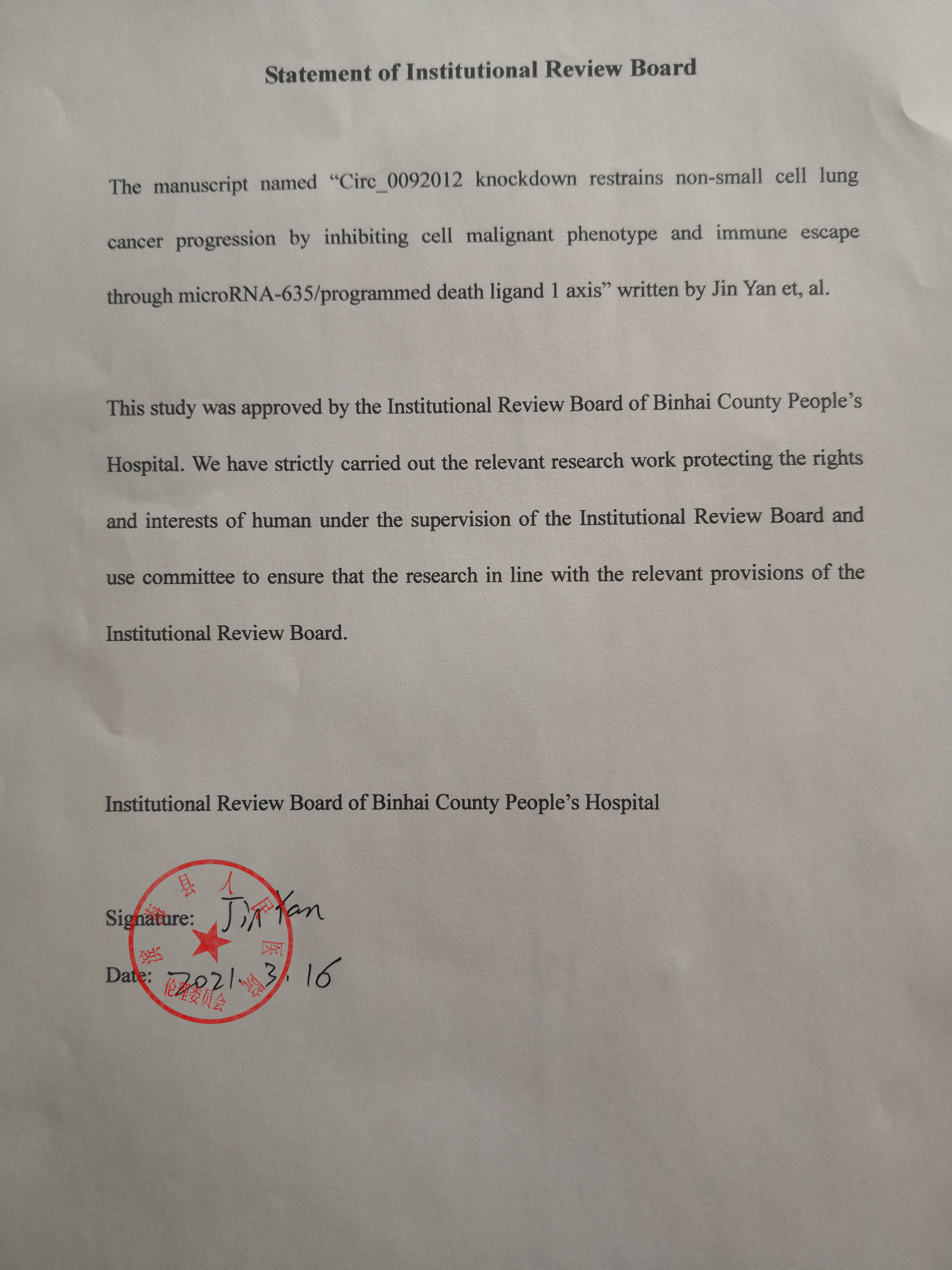

Supplement: Supplemental Material [file KBIE_A_2080386_SM5074.zip › IRB.jpg]
